# Effect of Cinnamon Tea on Postprandial Glucose Concentration

**DOI:** 10.1155/2015/913651

**Published:** 2015-07-14

**Authors:** Maria Alexandra Bernardo, Maria Leonor Silva, Elisabeth Santos, Margarida Maria Moncada, José Brito, Luis Proença, Jaipaul Singh, Maria Fernanda de Mesquita

**Affiliations:** ^1^Centro de Investigação Interdisciplinar Egas Moniz (CiiEM), Cooperativa de Ensino Superior Egas Moniz, Monte de Caparica, 2829-511 Caparica, Portugal; ^2^School of Forensic and Investigative Sciences and School of Pharmacy and Biomedical Sciences, University of Central Lancashire, Preston PR1 2HE, UK

## Abstract

Glycaemic control, in particular at postprandial period, has a key role in prevention of different diseases, including diabetes and cardiovascular events. Previous studies suggest that postprandial high blood glucose levels (BGL) can lead to an oxidative stress status, which is associated with metabolic alterations. Cinnamon powder has demonstrated a beneficial effect on postprandial glucose homeostasis in animals and human models. The purpose of this study is to investigate the effect of cinnamon tea (*C. burmannii*) on postprandial capillary blood glucose level on nondiabetic adults. Participants were given oral glucose tolerance test either with or without cinnamon tea in a randomized clinical trial. The data revealed that cinnamon tea administration slightly decreased postprandial BGL. Cinnamon tea ingestion also results in a significantly lower postprandial maximum glucose concentration and variation of maximum glucose concentration (*p* < 0.05). Chemical analysis showed that cinnamon tea has a high antioxidant capacity, which may be due to its polyphenol content. The present study provides evidence that cinnamon tea, obtained from *C. burmannii*, could be beneficial for controlling glucose metabolism in nondiabetic adults during postprandial period.

## 1. Introduction

Postprandial glucose level (PBG) has been reported to be an important factor in glycaemic control [1, 2]. The importance of postprandial glycaemia regulation is in accordance with epidemiological studies, which have demonstrated that postprandial hyperglycaemia is a predictor of diabetes and cardiovascular events [3, 4]. The postprandial state can stimulate the reactive oxygen species (ROS) production leading to an oxidative stress status. This status involves molecular mechanism for development of different complications associated with hyperglycaemia [5–7]. Moreover, postprandial oxidative stress can be accompanied by postprandial inflammation and endothelial dysfunction as reported by Ceriello et al. [8].

Postprandial glucose concentration refers to plasma glucose concentration after eating, which can be evaluated using a reference test standardized, 75 g oral glucose tolerance test (OGTT) [9]. During OGTT, plasma glucose level is obtained by secretion/action of insulin [10]. However, many other factors can also influence glucose metabolism including timing, quantity, and meal composition [11–13].

Many traditional plants and spices possess medicinal properties, such as control blood glucose levels. Cinnamon is one of these spices that has been demonstrated to be effective in improving glycaemia [14, 15] both in healthy and diabetic subjects. In healthy subjects, a study revealed that 5 g of cinnamon powder lowered PBG after OGTT [16] and another showed that 6 g added to a rice pudding improved PBG area under the curve (AUC) [17]. In type 2 diabetic subjects, cinnamon revealed that it can exert a hypoglycaemic effect, decreasing PBG and fasting blood glucose level (FBG) [18]. These beneficial effects seem to be related to its water-soluble polyphenols. An* in vitro* study showed that polyphenols possess an insulin-like action [19]. In addition, these cinnamon bioactive compounds revealed high antioxidant properties in human and animal models on oxidative stress through inhibiting lipid peroxidation [20, 21].

The aim of the present study is to investigate the effect of a cinnamon tea (6 g* C. burmannii*/100 mL) on postprandial capillary blood glucose level on nondiabetic adults.

## 2. Material and Methods

### 2.1. Subjects

Following ethical committee approval 30 nondiabetic adults with ages between 20 and 53 years were selected from the local community to participate in this study. A written informed consent was obtained from each volunteer after explaining the aim and experiment risk procedures. Inclusion criteria included subjects aged 18 or more, both genders with nondiabetic condition (fasting blood glucose level < 100 mg/dL [22]). Exclusion criteria comprised individuals who use medication for glycaemia control and have gastrointestinal symptoms or diseases. The study also excluded subjects who have altered medication, pregnancy, lactation, and allergy to cinnamon.

### 2.2. Study Design

Thirty nondiabetic adults were selected and randomly allocated in 2 groups (*n* = 15): control group, oral glucose tolerance test (OGTT_control_) alone, and intervention group, OGTT followed by cinnamon tea administration (OGTT_cinnamon_). The participants were asked not to ingest any cinnamon at the day before the intervention.

### 2.3. Subject Groups Characterization

At baseline (before interventions), general characteristic data, such as anthropometric data, medical condition, and pharmacological therapy, were collected using a questionnaire development by investigator. Participants were also questioned about usual cinnamon intake. A 24-hour dietary recall was taken preceding each intervention to compare food intake at the day before the intervention between groups. The* Food Processor SQL* (version 10.5.0) programme was used to analyse the nutritional composition of the food.

### 2.4. Oral Glucose Tolerance Test (OGTT)

The glucose (dextrose) was weighed (75 g) using an analytical balance and dissolved in 200 mL of water, according to American Dietetic Association [22]. Following overnight fasting (12 h) blood glucose level was measured using a capillary drop blood, before intervention (*t*_0_). In control group, subjects ingested glucose solution (200 mL) alone (OGTT_control_). In intervention group subjects ingested 100 mL cinnamon tea (OGTT_cinnamon_) immediately after glucose solution (200 mL) intake. Blood samples were collected, for each participant, at 30 (*t*_30_), 60 (*t*_60_), 90 (*t*_90_), and 120 (*t*_120_) minutes in control and intervention groups. Sterilized lancet, glucose meter equipment, and strips for glucose meter (*FreeStyle Abbott Diabetes Care*) were used for blood glucose level measurement.

### 2.5. Chemicals and Equipment for Antioxidant Capacity Studies

Ferric chloride (III) hexahydrate (FeCl_3_·6H_2_O), Folin-Ciocalteu (2,2-azinobis(3-ethylbenzothiazoline-6-sulfonic acid)), Trolox (6-hydroxy-2,5,7,8-tetramethylchroman-2-carboxylic acid), TPTZ 2,4,6-tri(2-pyridyl)-s-triazine, methanol (CH_3_OH), nicotinamide adenine dinucleotide (NADH), nitroblue tetrazolium (NBT) 2-amino-2-hydroxymethyl-propane-1,3-diol (tris), and phenazine methosulfate (PMS) were purchased from Sigma-Aldrich, gallic acid-1-hydrate (C_6_H_2_(OH)_3_COOH·H_2_O) was purchased from Acros Organics, and sodium carbonate (Na_2_CO_3_) was purchased from ICS Science group. All reagents were* pro analysis* grade.

All the absorbance measurements were performed in a Perkin-Elmer Lambda 25. The regents were weighed in an analytical balance (Sartorius, ±0.0001 g) and all the solutions were done with distilled water.

### 2.6. Cinnamon Tea Preparation

The* Cinnamomum burmannii* bark was purchased from Sucrame Company (Portugal) with Indonesia origin. Sticks of cinnamon (60 g) were soaked into 1000 mL of water. After 24 h at room temperature, cinnamon solution was heated for 30 min at 100°C and then filtered at room temperature. This method was adapted by Shen and coauthors [23]. After the cinnamon tea preparation a 100 mL individual dose was distributed to each participant. For chemical analysis, a hydromethanolic extract (50 : 50) was performed with cinnamon tea previously obtained.

### 2.7. Total Phenolic Content Determination

The total phenolic concentration in the extract was determined according to Folin-Ciocalteu method [24] employing gallic acid as standard. The results were expressed as mg for gallic acid equivalent (GAE)/g of extract. A volume of 375 *μ*L of cinnamon extract and 4 mL of sodium carbonate were added to 5 mL of Folin-Ciocalteu reagent. After 15 min the absorbance was measured at 765 nm. This test was performed for 8 replicates.

### 2.8. Antioxidant Assay Using Ferric Reducing Antioxidant Power (FRAP) Method

The method for determination of ferric reducing effect was based on the reduction, at low pH, employing a colourless ferric complex (Fe^3+^) to a blue-coloured ferrous complex (Fe^2+^) by electron-donating antioxidants action in 2,4,6-tri(2-pyridyl)-s-triazine (TPTZ) presence [25]. A fresh solution was prepared by mixing 25 mL of acetate buffer (300 mM, pH = 3.6) into 2.5 mL of TPTZ solution (10 mM) to HCl (40 mM) and 2.5 mL of FeCl_3_·6H_2_O solution (20 mM). The solution was heated at 37°C. Samples (150 *μ*L) were introduced in tubes with 2850 *μ*L of the FRAP solution and were maintained in the dark condition for 30 min. The absorbance was measured at 593 nm. Trolox (6-hydroxy-2,5,7,8-tetramethylchroman-2-carboxylic acid) was used as standard and the results were expressed in *μ*mol Trolox/L. This test was performed for 6 replicates.

### 2.9. Superoxide Anion Radicals Scavenging Activity (O_2_^∙−^)

Superoxide anion was generated by reacting phenazine methosulfate (PMS), nicotinamide adenine dinucleotide hydride (NADH), and oxygen causing reduced NBT in Formazan [26, 27]. A volume of 0.5 mL of sample was added to 2 mL of a solution containing NADH (189 *μ*M) and NBT (120 *μ*M) with Tris-HCl (40 mM, pH = 8). The reaction started after the addition of 0.5 mL of PMS (60 mM). Control sample was measured using only distilled water. After 5 min of incubation, control absorbance was measured at 560 nm at room temperature. Absorbance was measured for different concentrations of cinnamon samples in order to represent a curve of % of inhibition versus phenolic concentration. The percentage of superoxide anion inhibition was calculated using the following equation:

(1) $$\begin{array}{c} \%\;\;I=\frac{\text{Abs control}-\text{Abs corrected sample}}{\text{Abs control}}\times 100. \end{array}$$

### 2.10. Statistical Analysis

Statistical analysis was performed using Excel and SPSS Statistics (Statistical Package for Social Sciences) version 20.0 software for Windows. Data are presented as mean ± SD. Repeated Measures ANOVA of mixed type was used to assess the difference between the 2 groups for postprandial BGL at different times.

Independent samples *t*-test was used to assess the difference between the 2 groups for total caloric value, carbohydrates, protein and lipid, *C*_max⁡_, Δ*C*_max⁡_, and AUC_Incremental_ values.

All statistical tests were performed at the 5% level of significance.

## 3. Results

### 3.1. General Characteristics of Participants

The general characteristics of the study participants are shown in Table 1, reporting age, anthropometrics parameters, medical conditions, and pharmacologic therapy. The BMI data for both genders revealed that most of participants have regular weight [28]. Regarding body fat mass (FM) the results show that the sample subjects are also in normal range in both genders [28], but female had significantly more fat mass than male. In addition, female subject had significantly (*p* < 0.05) less muscular mass than male.

In what concerns the ingestion of total energy intake and macronutrient composition regarding carbohydrates, protein and lipid at the day before the intervention, the 2 groups can be considered homogeneous since they did not reveal significant differences (*p* > 0.05) (Table 2).

### 3.2. Postprandial Blood Glucose Level

Blood glucose levels (BGL) were measured for the 2 groups (OGTT_(control)_ and OGTT_(cinnamon)_) (Table 3). Statistical analysis revealed that there is no interaction between the independent and repeated measures factors (*p* = 0.209), which means that it is not possible to infer about differences in BGL in different moments.

**Figure 1:**
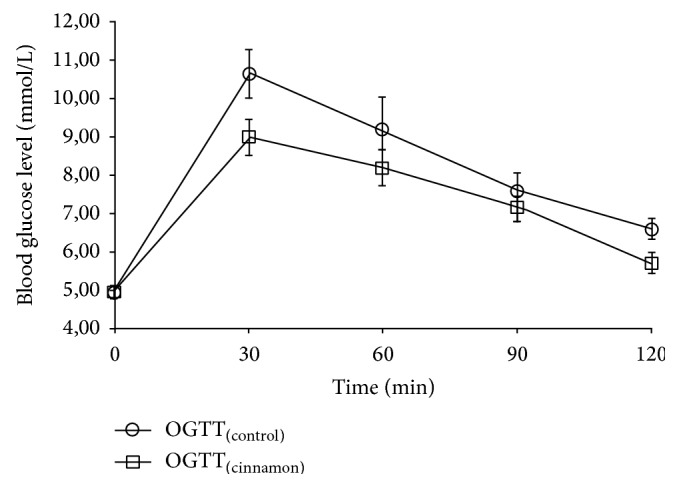
Mean (±SEM) time course of blood glucose levels (mmol/L) in nondiabetic subjects after OGTT_(control)_ (○) and OGTT_(cinnamon)_ (□).

However, the data showed that the administration of cinnamon tea after OGTT slightly decreased BGL mean values compared to OGTT in the absence of cinnamon intake (Figure 1).

The administration of cinnamon tea after OGTT resulted in a lower but not significantly postprandial blood glucose incremental area under the curve (AUCi) compared with OGTT_(control)_. However, the variation of maximum glucose concentration and maximum glucose concentration mean values were significantly lower (*p* < 0.05) in OGTT_(cinnamon)_ compared with OGTT_(control)_ (Table 4).

### 3.3. Total Phenol Content and Antioxidant Capacity

The data in Table 5 showed the total phenolic content, antioxidant capacity of cinnamon tea ingested by the participant. The superoxide anion radical scavenging activity (O_2_^∙−^) was measured at different concentration of* C. burmannii* tea. The results revealed that cinnamon tea has a strong inhibitory capacity, in a dose dependent manner, reaching 96% at 1143 mg/L gallic acid (half of the total phenols).

## 4. Discussion

Cinnamon capsule ingestion with either aqueous extract or cinnamon powder appears to improve fasting blood glucose level, independently of cinnamon species or extracts [15, 29]. Doubly linked polyphenol type-A polymers were identified, in the Ziegenfuss et al. study, as one of the possible bioactive compounds responsible for this effect [30]. In this study the administration of aqueous* C. burmannii *extract capsule (Cinnulin PF), with 1% of doubly linked polyphenol type-A polymers, improved fasting blood glucose levels, in prediabetes subjects. Moreover, in type 2 diabetic subjects or impaired fasting blood glucose, the administration of aqueous cinnamon extract also significantly reduced fasting blood glucose levels [31, 32].

In spite of consistent results regarding fasting blood glucose levels, the effect of cinnamon on postprandial glycaemia revealed heterogeneous results, which could be attributed to the bioactive compounds composition (which depends on extraction process, doses, species, and formulation), population samples, and study design employed in different studies [29].

The results of the present study demonstrated that cinnamon tea administration (6 g of* C. burmannii* into 100 mL water) slightly reduced PBG level after OGTT. The beneficial effects of this spice on glycaemia were reported after cinnamon powder ingestion where a significant reduction of PBG after 30 min of OGTT was observed [16, 17, 33]. However, Magistrelli and coauthors [33] showed no effect at 120 min after meal with cinnamon administration, compared with control meal. Other published data reported that cinnamon does not alter BGL at 120 minutes after OGTT [16, 17].

Although previous studies demonstrated that 3 g of cinnamon powder did not significantly alter AUC, *C*_max_ and Δ*C*_max_ BGL [34], the results from the present work showed that* C. burmannii* tea after OGTT significantly reduced *C*_max_ (*p* = 0.040) and Δ*C*_max_ (*p* = 0.029) compared with OGTT without cinnamon tea. This effect may be due to the high concentration employed in this study (6 g) compared with the other study, which uses 3 g.

Different molecular mechanisms have been suggested for the hypoglycaemic properties of this spice including reducing gastric empting [17], insulin-mimetic action [35, 36], which can lead to cellular glucose uptake [23], and reducing intestinal glycosidase activity. This effect on enzyme decreased breakdown of disaccharides into glucose, allowing a slow absorption of glucose and reducing PBG level [37].

The hypoglycaemic effect of cinnamon observed in the present study could also be attributed to the phenolic content of* C. burmannii* tea. According to literature, the molecular mechanism of action of cinnamon polyphenols includes the increase of insulin receptor-*β* protein in adipocytes suggesting acting beneficially in insulin signalling [35].

The data from this study suggest that the use of cinnamon tea can be beneficial to postprandial glucose levels; moreover its high phenolic content and antioxidant activity could also act beneficially. A significant relationship was found between antioxidant properties and total phenolic content in plants, suggesting that phenols are the bioactive compounds which contributed to their antioxidants activity [38]. In a study of Peng and coauthors, they show that proanthocyanidins of aqueous cinnamon extract can prevent the formation of advanced glycation-end product (AGE) [39]. AGE could be originated in high blood glucose levels conditions leading to reactive oxygen species production [5, 40]. Particularly, cinnamon tea employed in this study revealed a high activity of superoxide anion scavenging, which is in agreement with other published data demonstrating a strong scavenger capacity to free radicals* in vitro* models [41].

## 5. Conclusion

Data from this study provide evidence that cinnamon tea significantly decreased postprandial maximum glucose level in nondiabetic adults. The mechanism for cinnamon effect on glycaemia, based on slowing absorption of glucose through reducing intestinal glycosidase activity, cannot be applied to the present work since glucose solution was employed. One possible mechanism proposed to explain the effect of cinnamon tea on glycaemia may be related to the insulin action through the increasing of insulin receptor-*β* protein acting beneficially in insulin signalling. Further studies should be performed to investigate this mechanism.

## Figures and Tables

**Table 1:** Characteristics of the study participants (*n* = 30). Data represented mean (±SEM).

	Control group	Cinnamon group
	Mean (±SEM)	Mean (±SEM)
Age (years)		
Males	35.3 (±6.7)	30.2 (±3.7)
Females	38.3 (±3.5)	34.3 (±3.1)
BMI (Kg/m^2^)		
Males	23.6 (±1.2)	24.9 (±0.7)
Females	24.8 (±1.1)	24.1 (±0.7)
FM (%)		
Males	15.1 (±2.1)	17.2 (±0.8)
Females	27.6 (±1.7)	28.0 (±1.7)
MM (%)		
Males	52.7 (±2.1)	67.1 (±1.5)
Female	42.1 (±1.7)	41.8 (±1.3)

BMI: body mass index; FM: fat mass; MM: muscular mass.

**Table 2:** Dietary analysis of total energy intake (TEI), carbohydrates (CD), protein (P), and lipid (L) intake at the day before OGTT_(control)_ and at the day before OGTT_(cinnamon)_ by participants. Data are mean ± SEM; *n* = 15, each group.

Dietary Parameters	Day before OGTT_(control)_ Mean (±SEM)	Day before OGTT_(cinnamon)_ Mean (±SEM)	*p*
TEI (Kcal)	1708.01 (±97.04)	1736.51 (±113.74)	0.850
CD (g)	216.04 (±19.32)	225.40 (±15.33)	0.707
P (g)	75.66 (±6.15)	77.67 (±6.49)	0.823
L (g)	58.54 (±4.6)	58.47 (±6.61)	0.993

Independent sample *t*-test was used for statistical analysis.

**Table 3:** Mean blood glucose levels (mmol/L) obtained after oral glucose tolerance test (OGTT_(control)_) and after oral glucose tolerance test with cinnamon tea (OGTT_(cinnamon)_) at different moments: before OGTT (*t*_0_) and after 30 (*t*_30_), 60 (*t*_60_), 90 (*t*_90_), and 120 (*t*_120_) minutes. Data are mean ± SEM; *n* = 15, each group.

Time	OGTT_(control)_ Mean (±SEM) mmol/L	OGTT_(cinnamon)_ Mean (±SEM) mmol/L
*t*_0_	4.97 (±0.1)	4.99 (±0.1)
*t*_30_	10.14 (±0.4)	8.87 (±0.4)
*t*_60_	8.75 (±0.5)	8.24 (±0.4)
*t*_90_	7.66 (±0.5)	7.29 (±0.3)
*t*_120_	6.40 (±0.2)	5.86 (±0.2)

**Table 4:** Blood glucose level area under the curve (AUC), maximum glucose concentration (*C*_max⁡_), and variation of maximum glucose concentration (Δ*C*_max⁡_) in nondiabetic subjects at OGTT_(control)_ and OGTT_(cinnamon)_. Data are mean ± SEM (*n* = 15).

	OGTT_(control)_	OGTT_(cinnamon)_	*p*
	Mean (±SEM)	Mean (±SEM)	
	(mmol/L)	(mmol/L)	
AUCi (0–120 min)	403.73 (±48.5)	297.47 (±33.9)	0.084
*C*_max⁡_	10.63 (±0.6)	8.98 (±0.5)	0.040^*∗*^
Δ*C*_max⁡_	5.71 (±0.6)	4.0 (±0.5)	0.029^*∗*^

Independent samples *t*-test was assessed (^*∗*^differences for *p* < 0.05).

**Table 5:** Total phenolic content, antioxidant capacity of cinnamon tea. Values are mean ± SEM.

Chemical analysis	Mean (±SEM)
Total phenols (mg/L gallic acid, *n* = 8)	2286,3 (±48,0)
Antioxidant capacity: FRAP assay (*μ*mol Trolox/L, *n* = 6)	11853,4 (±322,8)
